# Histamine-Releasing Factor, a New Therapeutic Target in Allergic Diseases

**DOI:** 10.3390/cells8121515

**Published:** 2019-11-26

**Authors:** Yu Kawakami, Kazumi Kasakura, Toshiaki Kawakami

**Affiliations:** 1 Division of Cell Biology, La Jolla Institute for Immunology; La Jolla, CA 92037, USA; ykawakami@lji.org (Y.K.); kkasakura@lji.org (K.K.); 2 Department of Dermatology, School of Medicine, University of California San Diego, La Jolla, CA 92037, USA

**Keywords:** allergy, mast cells, basophils, IgE, FcεRI, HRF, translationally controlled tumor protein (TCTP)

## Abstract

Histamine-releasing activities on human basophils have been studied as potential allergy-causing agents for four decades. An IgE-dependent histamine-releasing factor (HRF) was recently shown to interact with a subset of immunoglobulins. Peptides or recombinant proteins that block the interactions between HRF and IgE have emerged as promising anti-allergic therapeutics, as administration of them prevented or ameliorated type 2 inflammation in animal models of allergic diseases such as asthma and food allergy. Basic and clinical studies support the notion that HRF amplifies IgE-mediated activation of mast cells and basophils. We discuss how secreted HRF promotes allergic inflammation in vitro and in vivo complex disease settings.

## 1. Introduction

Activation of mast cells and basophils via high-affinity IgE receptors (FcεRI) on the cell surface plays an essential role in allergic reactions. Multivalent allergens induce the aggregation or cross-linking of IgE-bound FcεRI to trigger their activation [1]. Activated mast cells and basophils release preformed chemicals (e.g., histamine, serotonin) and protein inflammatory mediators (e.g., proteases, tumor necrosis factor (TNF)), and de novo synthesize and secrete arachidonic acid-derived lipids, cytokines, chemokines, and growth factors [2,3]. These factors promote type 2 inflammation in allergic individuals. In this review, we will discuss histamine-releasing factor (HRF)-mediated regulation of mast cell/basophil activation via FcεRI and its roles in allergic and other immune diseases.

## 2. What Is HRF?

Cytokine-like factors able to activate basophils in body fluids of allergic patients have been studied for many years [4]. Several chemokines were shown to induce histamine release from human basophils in an IgE-independent manner [5,6,7]. On the other hand, an IgE-dependent factor with histamine-releasing activity (HRF) was molecularly cloned by Susan MacDonald’s group in 1995 [8]. Coincidentally, HRF happened to be identical to the protein termed translationally-controlled tumor protein (TCTP), fortilin, p21, and p23. It is often referred to as TCTP intracellularly and is required for cell cycle progression, proliferation, survival, and malignant transformation in a variety of cell types [9,10,11,12,13,14]. Extracellularly referred to as HRF (we follow this convention in this manuscript), it is an evolutionally conserved protein (96% identical between human and mouse proteins) composed of 172 amino acids with no known related proteins. Human HRF/TCTP is encoded by the *TPT1* gene on chromosome 13. Although numerous single nucleotide polymorphisms (SNPs) are associated with allergic diseases, no genetic associations with gene expression (eQTLs) are found in the *TPT1* locus (http://dicew-database.org). Similar to antigen/IgE-mediated activation, HRF induces not only histamine release, but also IL-4 and IL-13 secretion from human basophils and IL-13 and TNF secretion from murine mast cells [15,16]. Despite the lack of a signal sequence, it is secreted as a cargo of extracellular vesicles (EVs), particularly in exosomes [17,18,19,20]. Intriguingly, the responsiveness of basophils to HRF depends on a particular type of IgE; IgE derived from certain atopic patients, termed IgE^+^, can prime basophils in response to HRF, but other IgE molecules, termed IgE^−^, are unable to do so [21]. The dichotomy of IgE^+^ vs. IgE^−^ was discovered long before the molecular cloning of HRF, and several possibilities exist to explain the heterogeneity of IgE molecules: 1) structural differences in the constant regions of IgE, for example, by differences in glycosylation or alternative mRNA splicing at the ε chain 3′ terminal region [22]; 2) IgE^+^ being an HRF-specific IgE antibody, that is, HRF acting as an IgE autoantigen; 3) IgE^+^ reactivity due to the presence of anti-IgE antibodies in the serum.

In contrast to an earlier report suggesting that HRF does not bind to IgE [23], Kashiwakura et al. showed that a subset of IgE and IgG molecules are able to directly bind to HRF via two Ig Fab-interacting sites: the N-terminal 19 residue stretch (N19) and the H3 helix [24]. These observations are in line with an earlier speculation that the dichotomy of IgE^+^ vs. IgE^−^ may be caused by differences in IgE variable region subgroups [25]. However, another speculation that IgE^+^ reactivity is related to glycosylation of IgE [21] was not supported by the observation that mannose-specific lectins could not distinguish between basophils sensitized with IgE^+^ or with IgE^−^ [26]. Despite these studies, it still remains possible that glycosylation at V_H_ and V_L_ regions might contribute to the IgE^+^ reactivity. In light of recent revelations regarding IgE glycosylation [27], the potential role of glycosylation may be worth revisiting.

## 3. Bioactive Forms of HRF

**Figure 1:**
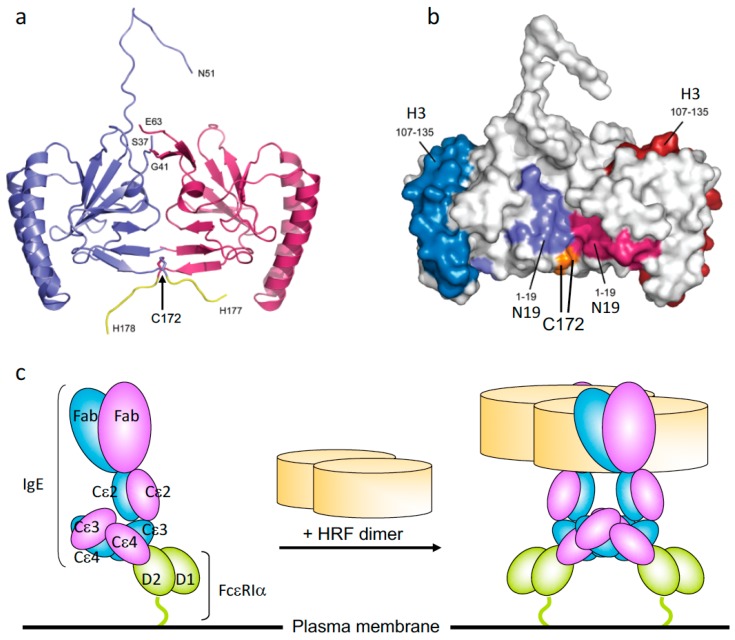
The crystal structure of histamine-releasing factor (HRF) dimer and HRF dimer/IgE-mediated FcεRI crosslinking. (**a**) Overall structure of a human HRF dimer. The two molecules of the asymmetric unit are colored blue and pink. The C-terminal tag is colored yellow, and the positions of C-terminal residues and residues adjacent to the disordered loop are indicated. (**b**) The two monomers of the HRF dimer are colored white and Cys172 is colored orange. For the first monomer, the two IgE binding sites, mapped to residues Met1–Lys19 (N19), and Arg107–Ile135 (H3), are colored light blue and dark blue, respectively. For the second monomer, residues 1-19 (N19) and 107-135 (H3) are colored light and dark pink, respectively. (**c**) Model for HRF dimer/IgE-mediated FcεRI crosslinking. IgE binds FcεRI α chain via the interaction between IgE–Cε3 and FcεRIα–D2 domains. One HRF molecule can bind one (this version depicted) or two molecules of IgE via interactions with the N19 and H3 regions of HRF. After binding of an HRF dimer, two (this version depicted) or four FcεRI α chain-nucleated complexes will be formed (Right). The cytoplasmic portion of FcεRI α as well as β and γ chains of FcεRI are omitted for clarity.

HRF is constitutively secreted as a monomer, a disulfide-linked dimer, and higher molecular weight oligomers. Crystal structures of HRF monomers from various species and a homodimer of human HRF have been solved. The homodimer is made by a disulfide bond through a Cys172-Cys172 linkage between two monomers [28,29]. Kim et al. showed that N-terminally truncated recombinant rat HRF proteins, Del-N11TCTP and Del-N35TCTP, but not full-length TCTP, also form disulfide-linked dimers with strong cytokine-like activity [29]. However, Doré et al. observed dimers of full-length mouse and human HRFs [28]. Consistent with the efficacy of HRF inhibitors in allergic disease models (see below), IgE-binding sequences (i.e., N19 and H3) are exposed on the molecular surface of HRF dimer (Figure 1a,b) [28]. Recombinant HRF homodimers, but not monomers, synthesized in *E. coli* can activate murine mast cells [30]. GST-HRF fusion proteins induce not only histamine release [8] but also secretion of IL-4 and IL-13 from human basophils [15,16]. It is well known that GST fusion proteins can form dimers. Thus, these results suggest that FcεRI-bound IgE molecules are cross-linked by HRF dimers (Figure 1c). HRF homodimers are also able to enhance IgE and antigen-stimulated production of IL-6, IL-13, and TNF but not β-hexosaminidase release (which is fully activated by stimulation with antigen) from murine mast cells. This result suggests that cytokine production requires stronger and/or more persistent FcεRI cross-linking than does degranulation. These observations can be extended to the argument that HRF exerts its effects by activating FcεRI signaling pathways. However, subtle differences in signaling may occur, as components of the ligand complex are different when cells are stimulated with antigen/IgE complexes bound to FcεRI with or without HRF. Intranasal instillation of recombinant HRF (including HRF dimers), but not HRF-2CA (a monomeric mutant of HRF with the two cysteine residues being replaced with alanine), reduced/carboxymethylated or boiled HRF, in naïve mice triggered airway inflammation in an FcεRI-dependent manner [24]. The wide gamut of signs seen in allergic diseases ranging from the mild skin rashes and gastrointestinal symptoms, to more severe signs such as pulmonary distress and systemic anaphylaxis, could be due to different levels of contributions of HRF dimer/oligomers as well as other factors such as variable antigen valencies and concentrations or FcεRI occupancy by antigen-specific IgE. Further analysis of HRF regulation of FcεRI activation is warranted to understand how different forms of HRF affect allergen/IgE-mediated FcεRI cross-linking.

## 4. HRF in Allergic and Immune Diseases

Allergic diseases such as atopic dermatitis, food allergy, asthma, and allergic rhinitis are type 2 inflammatory diseases in allergen-sensitized individuals with organ-specific or systemic disease susceptibility [31,32,33]. Type 2 inflammation is caused by type 2 innate lymphoid cells, allergen-specific Th2 cells, and epithelial-derived cytokine- and Th2 cytokine-recruited mast cells and eosinophils [34,35,36,37]. HRF secretion was found in nasal, skin blister, and bronchoalveolar lavage fluids during the late phase of allergic reactions [38], implicating HRF in allergic diseases (Table 1). Long before the molecular nature of HRF was revealed, a study showed that patients with food allergy and atopic dermatitis, but not patients with atopic dermatitis alone, have higher rates of spontaneous release of histamine from basophils than normal subjects [39], implying HRF’s involvement in food allergy. However, definitive evidence for pathological roles of HRF in allergy had been elusive until recently, as there were intractable obstacles in HRF research: (i) HRF/TCTP has both intracellular and extracellular functions, but no tools were available to dissect these functions in complex in vivo settings. (ii) Despite considerable efforts, researchers were unable to identify an HRF receptor for many years [23]. (iii) HRF knockout mice were embryonically lethal [40,41,42], thus severely limiting in vivo functional studies. As described above, Kashiwakura et al. identified a subset of IgE and IgG molecules as HRF receptors [24]: mapping of the Ig Fab-binding

**Figure 2:**
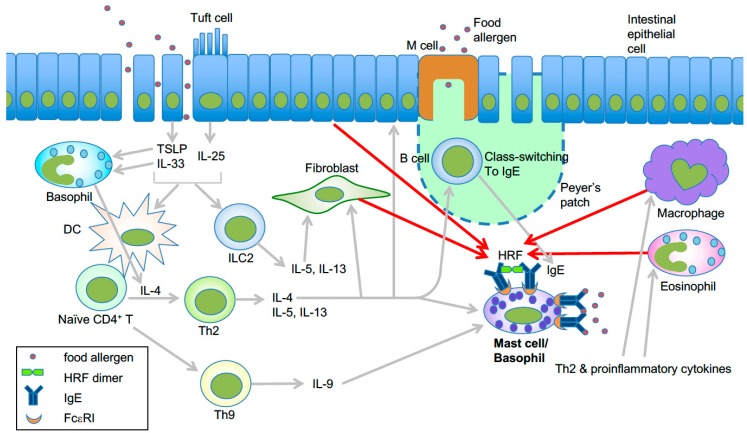
Model of HRF-mediated amplification of type 2 inflammation in food allergy. Epithelial damage or inflammation in the gut promotes increased entry of food allergens and secretion of the epithelial cytokines TSLP, IL-25, and IL-33 [44]. These cytokines initiate a Th2-skewed immune response. TSLP can enhance OX40L expression in dendritic cells, which induce Th2 cell differentiation of naïve CD4^+^ T cells [45]. IL-25 secreted by tuft cells may help the expansion of type 2 innate lymphoid cells (ILC2) [46]. Th2 cells along with ILC2 cells promote the Th2 cell-mediated immune response, which includes IgE class switch recombination in B cells, eosinophil accumulation, and mastocytosis. IL-9 promotes the expansion of IL-9-producing mucosal mast cells [47]. Basophils are also required for production of antigen-specific IgE as well as oral allergen-induced food allergy during sensitization [48,49] and allergen challenge phases [50]. IL-4 derived from basophils stimulated by cytokines such as IL-33 seems to be required for Th2 cell differentiation [51], and IL-4 promotes intestinal mast cell accumulation and activation [52]. HRF dimer/oligomers secreted from several types of cells amplify intestinal inflammation by enhancing antigen/IgE-mediated activation of mast cells and basophils [30]. This is likely due to increased HRF secretion by several types of cells in response to Th2, proinflammatory and epithelial cytokines. Modified from ref. 66 with permission from the journal *Allergy*.

Sites within the HRF molecule led to the discovery of HRF sequence-based competitive inhibitors, N19 and H3 peptides, as well as a monomeric mutant HRF-2CA, all of which blocked HRF–Ig interactions without affecting intracellular functions of TCTP. Administration of these inhibitors drastically reduced type 2 inflammation in mast cell-dependent murine models of atopic asthma and immediate hypersensitivity of the skin. Intranasal administration of recombinant HRF into naïve mice caused lung inflammation in an FcεRI and mast cell-dependent manner [24]. Thus, this study in 2012 solved several major questions about HRF, including the aforementioned issues (i) and (ii). More recently, Ando et al. showed that HRF dimers, but not monomers, are able to activate HRF-reactive IgE-bound mast cells and basophils [30]. Intragastric administration of HRF inhibitors, which preferentially targeted mast cells in the small intestine, strongly reduced diarrhea occurrence, intestinal inflammation, and systemic anaphylaxis in a murine model of food allergy [30,43]. Levels of HRF oligomers (including dimers) in the small intestine and HRF-reactive IgE in serum were increased in food allergic mice, but HRF oligomers were decreased by HRF inhibitors. Patients with egg allergy also had higher blood levels of HRF-reactive IgE, and successful oral immunotherapy led to reduced HRF-reactive IgE. Thus, these data suggest that in allergen-sensitized mice, secreted HRF oligomers bind to the Fab portion of IgE and reduce the threshold of allergen concentrations required to crosslink IgE-bound FcεRI to activate intestinal mast cells and basophils to elicit the food allergy phenotype (Figure 2).

Another interesting drug candidate is a 7-mer peptide, called dTBP2. It was identified by phage display as a peptide more strongly bound to HRF dimer than to monomeric HRF [53]. dTBP2 ameliorated ovalbumin-induced airway inflammation in mice and reduced IL-8 release from BEAS-2B human bronchial epithelial cells. Recently, dehydrocostus lactone, a sesquiterpene from *Saussurea lappa Clarke*, which is able to bind to HRF dimers, was reported to suppress ovalbumin-induced airway inflammation [54]. However, given its action on various biological activities, it is premature to conclude that the anti-inflammatory effects of this compound are due to the inhibition of HRF dimer.

Atopic dermatitis is a heterogeneous disease in terms of the pathogenic role of the IgE–FcεRI axis [55,56]. Interestingly, atopic dermatitis patients have increased levels of HRF, and some patients have higher levels of HRF-reactive IgE compared to healthy individuals [57]. Polyclonal IgE molecules present in sera from atopic dermatitis patients activated mast cells [58], similar to highly cytokinergic IgE [59]. Topical administration of dTBP2 reduced allergen-induced atopic dermatitis in NC/Nga mice [60], a murine model of atopic dermatitis [56].

Chronic idiopathic urticaria (CIU) or chronic spontaneous urticaria is a disease of itchy red skin or skin colored hives with no known cause lasting for six weeks or more. IgG autoantibodies against IgE or FcεRI may contribute to CIU pathogenesis in 30%–40% of the patients [61]. Activation of skin mast cells plays a key role in skin inflammation of CIU. Interestingly, a recent study reported increased serum levels of both HRF and HRF-reactive IgE in CIU patients compared to healthy cohorts, and there was a linear correlation between HRF and HRF-reactive IgE concentrations in CIU patients [62]. Furthermore, the HRF-reactive IgE level was correlated with disease severity. The authors observed degranulation in the human mast cell line LAD-2 sensitized with serum of a CIU patient and stimulated with HRF. They suggested that synergistic actions of HRF and HRF-reactive IgE may play an important role in the CIU pathogenesis.

Pulmonary arterial hypertension (PAH) is a rare, but often lethal disease characterized by a sustained increase in pulmonary arterial pressure and severe vascular remodeling. Heritable PAH commonly involves mutations in bone morphogenetic protein receptor type II (*BMPR2*). Excessive proliferation of pulmonary vascular endothelial cells is seen in this disease caused by an imbalance between cell proliferation and apoptosis. Increased plasma and lung levels of HRF associated with exosomes derived from endothelial cells were found in PAH patients compared to normal subjects [63,64]. The exosome-derived HRF was taken up by pulmonary artery smooth muscle cells in in vitro co-cultures, and promoted proliferation and suppressed apoptosis of the latter cells [20,63]. These results suggest that HRF may not require a specific cell surface receptor for this type of intercellular communication, as extracellular HRF that has reached the interior of recipient cells would interact with its target molecules, potentially including Bcl-XL and Mcl-1. Interestingly, essentially all exosome-associated (and microparticle-associated) HRF in endothelial cells was dimeric [63]. However, there is no evidence that the function of intracellular TCTP molecules is operated by the dimeric form, as the vast majority of intracellular TCTP molecules is monomeric [30]. No definitive studies have been conducted to assign the functions of HRF/TCTP to either its monomeric or dimeric forms (or other forms) in PAH and other diseases.

## 5. Concluding Remarks

It is not easy to assign a particular pathogenic role to the secreted HRF molecules separate from the intracellular TCTP molecules. Targeting HRF is a promising approach toward prevention of allergic diseases such as food allergy and asthma [24,30,65]. However, all of the current HRF inhibitors have yet to be fully characterized as therapeutic agents. It is highly desirable to gain both pharmacological and genetic evidence before the field moves to clinical trials of candidate HRF inhibitors. However, genetic studies without affecting the function of intracellular TCTP are difficult if an experiment is conducted with TCTP conditional knockout (CKO) mice, including inducible CKO mice [40,66]. It is likely that the targeted cells may die because of their dependence of survival on TCTP. With such limitations, RNA interference (siRNA or shRNA) may be better suited to in vitro and in vivo experiments [66]. An alternative approach is to use heterozygous TCTP KO mice. Indeed, Pinkaew et al. showed that atherosclerotic lesions in TCTP^+/−^Ldlr^−/−^Apobec1^−/−^ mice contain fewer macrophages and more apoptotic cells compared to TCTP^+/+^Ldlr^−/−^Apobec1^−/−^ mice [67]. Transgenic overexpression may also be useful for analysis of HRF. Yeh et al. generated an inducible transgenic mouse model with HRF targeted to lung epithelial Clara cells [68]. They showed that HRF exacerbates the allergic asthmatic responses, although it is not clear whether secreted HRF was responsible for the worsened phenotype. Despite these obstacles, HRF inhibitors may be a promising approach toward preventing or treating food allergy and other IgE/HRF-dependent allergic diseases.

## Figures and Tables

**Table 1:** HRF in allergic and immune disorders.

Disease	Modulation of Animal Disease Models by HRF or HRF Inhibitors	Human Patients
**Asthma**	↓OVA-induced airway inflammation by HRF inhibitors (N19, H3) ↓*Aspergillus fumigatus*-induced airway inflammation by HRF inhibitors (N19) ↑airway inflammation induced by intranasal instillation of recombinant HRF ↓OVA-induced airway inflammation by dTBP2 peptide	
**Atopic dermatitis (AD)**	↓passive cutaneous anaphylaxis by HRF inhibitors (N19) ↓house dust mite allergen-induced skin inflammation in NC/Nga mice by dTBP2 peptide	↑serum HRF, ↑serum HRF-reactive IgE
**Food allergy (FA)**	**OVA-induced FA:** ↑serum HRF-reactive IgE, ↑HRF dimer/ oligomers in jejunum, ↑diarrhea, ↑hypothermia, ↓physical activity, which were all reduced by HRF inhibitors (N19, HRF-2CA)	**Egg allergy:** ↑serum HRF-reactive IgE, which was reduced by successful OIT^1^
**Chronic idiopathic urticaria (CIU)**		↑serum HRF, ↑serum HRF-reactive IgE
**Pulmonary arterial hypertension (PAH)**		↑plasma and lung HRF associated with exosomes

Oral immunotherapy (OIT^1^). ↓, decreased; ↑, increased.
